# Associations between changes in precerebral blood flow and cerebral oximetry in the lower body negative pressure model of hypovolemia in healthy volunteers

**DOI:** 10.1371/journal.pone.0219154

**Published:** 2019-06-28

**Authors:** Jonny Hisdal, Svein Aslak Landsverk, Ingrid Elise Hoff, Ove Andreas Hagen, Knut Arvid Kirkebøen, Lars Øivind Høiseth

**Affiliations:** 1 Section of Vascular Investigations, Department of Vascular Surgery, Division of Cardiovascular and Pulmonary Diseases, Oslo University Hospital, Oslo, Norway; 2 Faculty of Medicine, University of Oslo, Oslo, Norway; 3 Department of Anesthesiology, Division of Emergencies and Critical Care, Oslo University Hospital, Oslo, Norway; 4 Norwegian Air Ambulance Foundation, Oslo, Norway; University of Bern, University Hospital Bern, SWITZERLAND

## Abstract

Reductions in cerebral oxygen saturation (ScO_2_) measured by near infra-red spectroscopy have been found during compensated hypovolemia in the lower body negative pressure (LBNP)-model, which may reflect reduced cerebral blood flow. However, ScO_2_ may also be contaminated from extracranial (scalp) tissues, mainly supplied by the external carotid artery (ECA), and it is possible that a ScO_2_ reduction during hypovolemia is caused by reduced scalp, and not cerebral, blood flow. The aim of the present study was to explore the associations between blood flow in precerebral arteries and ScO_2_ during LBNP-induced hypovolemia. Twenty healthy volunteers were exposed to LBNP 20, 40, 60 and 80 mmHg. Blood flow in the internal carotid artery (ICA), ECA and vertebral artery (VA) was measured by Doppler ultrasound. Stroke volume for calculating cardiac output was measured by suprasternal Doppler. Associations of changes within subjects were examined using linear mixed-effects regression models. LBNP reduced cardiac output, ScO_2_ and ICA and ECA blood flow. Changes in flow in both ICA and ECA were associated with changes in ScO_2_ and cardiac output. Flow in the VA did not change during LBNP and changes in VA flow were not associated with changes in ScO_2_ or cardiac output. During experimental compensated hypovolemia in healthy, conscious subjects, a reduced ScO_2_ may thus reflect a reduction in both cerebral and extracranial blood flow.

## Introduction

Cerebral blood flow (CBF) has generally been assumed to be autoregulated [1], meaning that CBF is maintained as long as mean arterial pressure (MAP) is kept between a lower and upper limit. However, cerebral blood flow regulation is complex, and studies have shown that cardiac output may affect CBF independently of MAP [2–5].

During hypovolemia in conscious humans, reduced stroke volume is initially compensated by increased systemic vascular resistance and heart rate, maintaining MAP. This makes clinical signs and symptoms subtle [6] and the compensated phase of volume loss difficult to detect. Thus, methods to better diagnose compensated hypovolemia in conscious, unanesthetised patients, e.g. trauma patients in a prehospital environment, are needed.

Cerebral oxygen saturation (ScO_2_) can be estimated by near infra-red spectroscopy (NIRS), and is believed to reflect the balance between oxygen delivery and utilisation in the region where the measurement is performed [7]. When estimating ScO_2_ with NIRS, the light has to pass extracranial tissues which may influence the results, and the devices typically attempt to filter out any influence by these tissues. Despite these attempts, it has been shown in several models that scalp blood flow, mainly supplied by the external carotid artery (ECA), may contaminate the NIRS-derived estimates of cerebral oxygenation [8–10]. The degree to which this contamination occurs may be device and algorithm specific [9]. During hypovolemia, blood flow is typically diverted away from the skin towards more highly prioritized organs.

Lower body negative pressure (LBNP) is a model of central hypovolemia induced by pooling of blood in the lower body and thereby reduced preload [11, 12]. Reductions in ScO_2_ when exposing healthy volunteers to LBNP have been found previously [13], leading to the question whether ScO_2_ can be used as a diagnostic tool to detect compensated hypovolemia. Based on the premises that 1) cerebral blood flow is reduced with hypovolemia, 2) ScO_2_ is reduced with hypovolemia and 3) a reduced ScO_2_ may be caused by extracranial contamination; to the best of our knowledge, it remains to be elucidated if reduced ScO_2_ during compensated hypovolemia reflects reduced cerebral blood flow or merely extracranial contamination. As extracranial tissues may be more susceptible to disturbances by e.g. pain and temperature, this may be of importance both pathophysiologically as well as for the potential ability of ScO_2_ to be used clinically to diagnose compensated hypovolemia in conscious, unanesthetised patients.

The aim of the present study was therefore to explore if changes in ScO_2_ during hypovolemia in the LBNP-model were associated with changes in flow in the internal carotid artery (ICA). In addition, we also explored the associations between ScO_2_ and flow in the ECA, vertebral artery (VA), cardiac output and scalp skin blood flow.

## Materials and methods

### Subjects

The study was approved by the Regional Committees for Medical and Health Research Ethics (REC South East D, reference 2015/344). After written and oral informed consent, 20 healthy adults (age >18 years) volunteers were included between November 2015 and December 2016. Exclusion criteria were: 1) disease or condition reducing exertional capacity or requiring regular medication, allergy excepted; 2) previous syncope, presumed vasovagal syncope excepted; 3) known cardiac arrhythmia; 4) pregnancy. The subjects were allowed light meals and could drink freely, but refrained from caffeine, nicotine and strenuous physical activity on the day of the experiment. The subjects were familiarized with the LBNP-model and measurement techniques, and a focused medical history and physical examination was performed.

### Study protocol

Before the experiment, subjects were randomised (block size six) to have performed three consecutive measurements of ICA, ECA or VA flow to calculate precision. The ultrasound probe was removed from the skin and replaced between the three measurements to approach the repositioning of the probe necessary during the LBNP-sequence when measuring the three different arteries.

**Fig 1:**
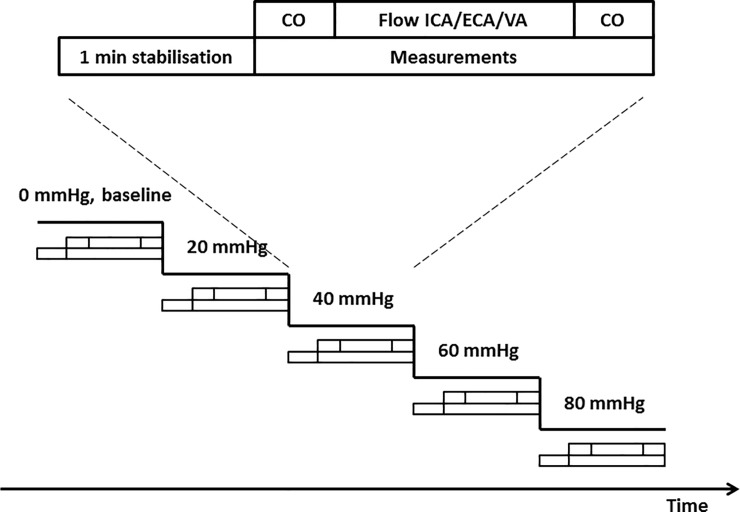
Experimental protocol. After 1 min stabilisation at each LBNP-level, cardiac output (CO) was measured. Flow in ICA, ECA and VA were measured thereafter in alternating order before measuring cardiac output again.

Measurements were performed at baseline and LBNP 20, 40, 60 and 80 mmHg. LBNP was released and the experiment ended earlier at the subjects´ request or if they displayed symptoms or signs of impending circulatory collapse (drop in heart rate or MAP, nausea or lightheadedness). Only completed LBNP-levels were used, i.e. the level at which decompensation occurred was removed. The study protocol is illustrated in Fig 1. After 1 min stabilisation, cardiac output was calculated over at least one respiratory cycle. Thereafter, ICA, ECA and VA-flow were measured, each over at least one respiratory cycle. The order of these three measurements alternated within subjects for each LBNP-level. The subjects were also randomised in blocks of six for which of the measurements were performed first at baseline (thus also changing the order in the subsequent LBNP-levels). Lastly, cardiac output was calculated again. Cardiac output calculations obtained before and after carotid/vertebral flow measurements were averaged as the value for that LBNP-level.

### LBNP, measurements and data processing

The experiments were performed between 8:00 AM and 4:00 PM in a vascular investigation lab air-conditioned to 20–22°C. Subjects were placed in the LBNP-chamber sealed with a neoprene skirt just above the iliac crest. Two NIRS sensors (Adult SomaSensor; Covidien, Mansfield, MA, USA; Invos 5100C cerebral/somatic oximeter; Somanetics, Troy, MI, USA) were placed on the right and left forehead. Data were extracted from the serial output every 7–8 s to software in LabVIEW 14.0 (National Instruments, Austin, TX, USA).

Flow in the right ICA, ECA and VA were measured over at least one respiratory cycle using a linear 9 MHz ultrasound probe of a Vivid E9 ultrasound machine (GE Vingmed Ultrasound, Horten, Norway), all measurements performed by the same experienced operator. Although the probe had to be moved between measurements of the three different arteries, we attempted to place the Doppler sampling volume for each artery on the same anatomic location. The carotid bifurcation was used as a landmark for the ICA and ECA measurements, placing the sampling volume 1.5–2 cm distal to the bifurcation. For VA-measurements, the vertebral interspace with the most prominent color Doppler signal was used. When measuring cerebral blood flow using this method, repeatability comparable to reference techniques has been found [14]. The velocity-time integrals were calculated using the automatic border detection algorithm of the ultrasound machine and flow by multiplying this integral by the cross-sectional area measured at LBNP 0 mmHg (baseline).

**Fig 2:**
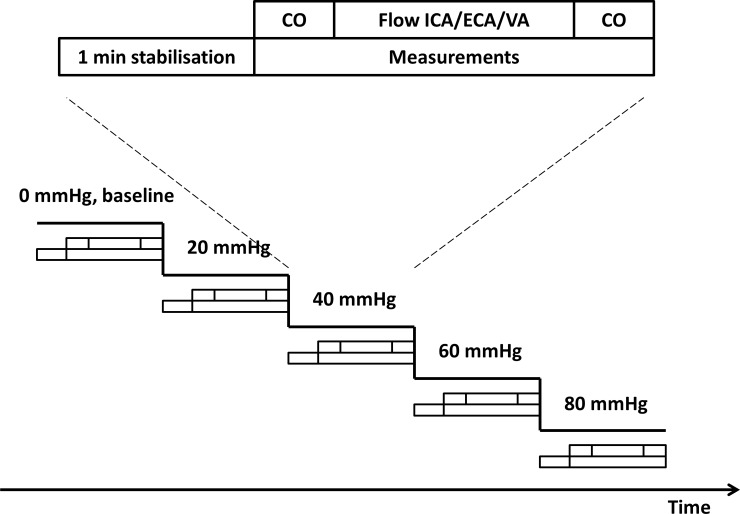
Measurements. Schematic overview of the performed measurements.

Stroke volume was estimated using a 2 MHz suprasternal Doppler probe (SD-50; Vingmed Ultrasound), assuming a diameter of the aortic orifice of 20 mm and an angle to the blood flow of 20° [15]. Arterial blood pressure was measured using a photoplethysmographic volume-clamp method (Finometer; FMS Finapres Measurement Systems, Arnhem, The Netherlands) around the middle left finger. A 5-lead ECG and heart rate were acquired (Marquette Solar 8000i and TramRacA4; GE Medical Systems, Milwaukee, WI, USA). Scalp skin blood flow was estimated with two laser Doppler flowmetry (LDF) probes (PeriFlux 4001 Master; Perimed AB, Järfälla, Sweden). One probe was attached to the forehead above the NIRS-sensor on the left side (immediately below the hairline) and the other below the NIRS-sensor on the right side (immediately above the eyebrow). SpO_2_ and peripheral (finger) perfusion index was measured using a Masimo Radical 7 pulse oximeter, software 7.3.1.1, (Masimo Corp., Irvine, CA, USA) and exported at 0.5 Hz by the TrendCom software (Masimo Corp.). Acral skin blood flow was measured with a LDF probe attached to the left thumb (Perimed AB). End-tidal CO_2_ (EtCO_2_) was measured using a sidestream capnograph (Cap10; Medlab medizinische Diagnosegeräte GmbH, Stutensee, Germany) with gas sampling from a face mask offering minimal resistance to air flow and added dead space. Expired CO_2_-concentration was exported by the analog output of the capnograph. A schematic overview of the measurements is presented in Fig 2.

Analog data for aortic blood flow velocity, ECG, LDF and MAP were sampled in Regist3 software (Morten Eriksen, University of Oslo, Oslo, Norway) and SignalExpress Version 14.0.0 (National Instruments) at 300 and 400 Hz, respectively, exported as text files and handled in R 3.4.0 (R Foundation for Statistical Computing, Vienna, Austria) using RStudio 1.0.143 (RStudio, Boston, MA, USA). After time-synchronization of the different signals, velocity-time integrals of the aortic Doppler were calculated gated by the R-peaks of the ECG. Stroke volume was calculated from the velocity-time integrals, giving cardiac output by multiplying with heart rate. The other data were averaged over 1 s. LDF measurements and perfusion index were log_e_-transformed for further analysis due to right-skewness of data, as judged by visual inspection of histograms and QQ-plots. Data were plotted, and obviously erroneous values due to disturbances, e.g. motion artefacts, were removed. After removing the first 1 min of each LBNP-level (stabilisation), trimmed means, trimming the 5% highest and lowest values, were calculated for each LBNP-level. Respiratory rate was calculated from the capnography curve.

### Statistics

To describe single variables through the experiment and account for dependency of observations within subjects, LBNP-level was treated as a factor in mixed-effects regression models with subject as a random effect, using the “nlme” [16] package in R. Confidence intervals and comparisons to baseline (LBNP 0) were performed using the glht-function of the “multcomp” package in R, with “single-step” adjustment of p values due to multiple comparisons [17]. To examine within-subject associations of changes between variables, regression analyses were performed with the difference between each observation and that subject´s mean value as predictors (centred values, on x-axes in the plots) [18]. In the scatterplots, the coefficient with confidence interval and p value for the slope is presented. For visual clarity, the data for the dependent variables (y-axes) are also centred in the plots. P values <0.05 were considered statistically significant. Values are mean (SD) or median (25^th^ - 75^th^-percentiles). Precision was calculated as 1.96 × √(residual mean square) in a one-way ANOVA with subjects as factors [19]. Analyses were performed in R using RStudio, figures made in SigmaPlot 12.5 (Systat Software, San Jose, CA, USA).

We hypothesised that there would be an association between changes in ScO_2_ and the ICA blood flow during increasing LBNP. To determine sample size, a within-subject correlation coefficient of 0.4 between ScO_2_ and the ICA-flow was assumed. Based on simulations in an ANCOVA with α = 0.05 and 1-β = 0.9, a statistically significant association could be found with 15 subjects assuming all subjects completing all 5 LBNP-levels. As we expected some subjects to complete the protocol before reaching LBNP 80 mmHg, we included 20 subjects.

## Results

### Hemodynamics

20 subjects (11 males, 9 females), age 25 (22–27) years, height 176 (9) cm and weight 69 (8) kg were included. Nine subjects completed LBNP 80 mmHg without hemodynamic collapse. Nine subjects completed LBNP 60 mmHg and two subjects completed LBNP 40 mmHg before ending the experiment. The experiment lasted 19 (18–20) min with each LBNP-level lasting 4.0 (3.7–4.5) min.

**Fig 3:**
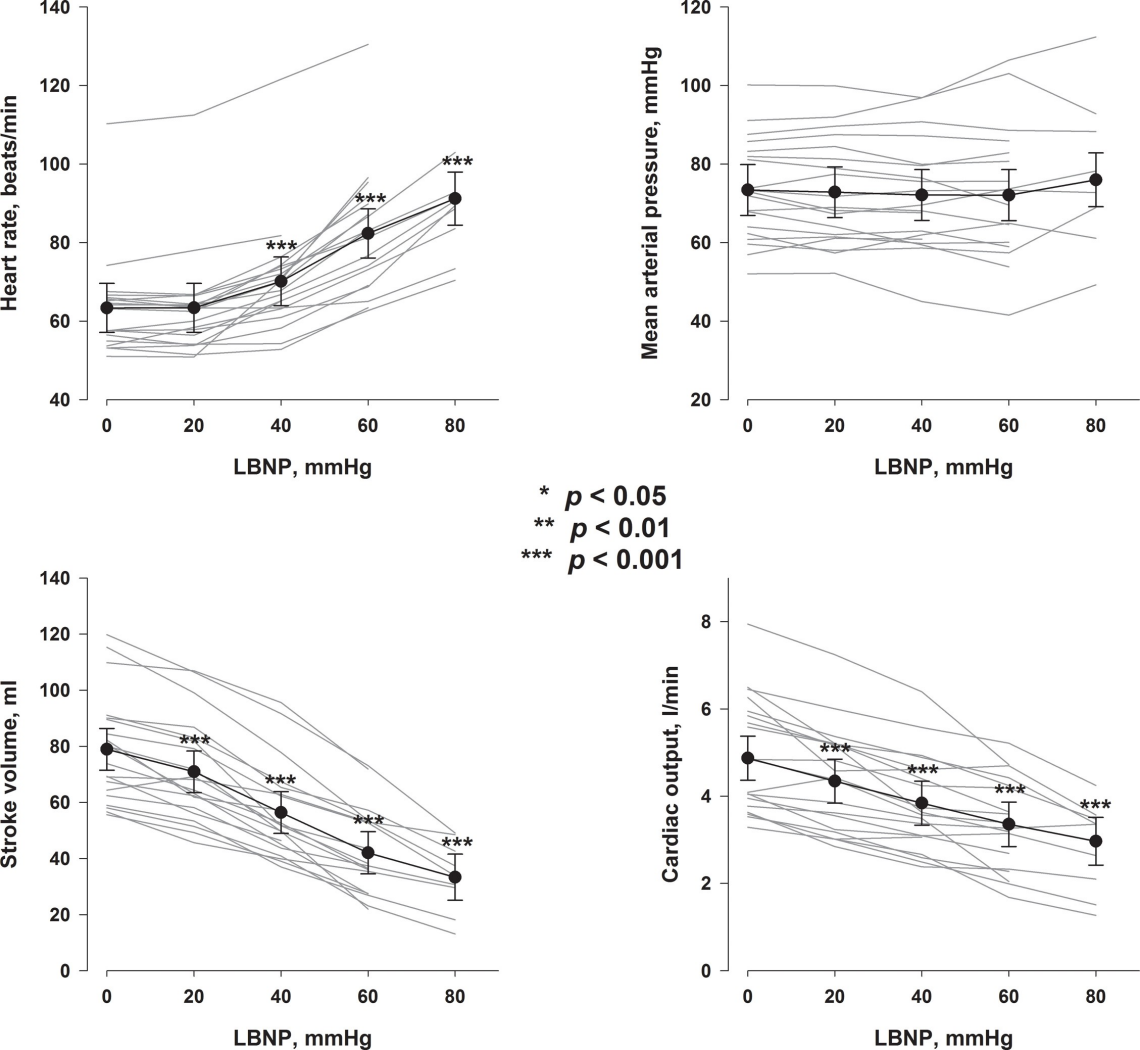
Hemodynamic variables during the experiment. Grey lines are values for each subject. Black symbols are estimates with 95% CI for each LBNP-level. P values are for comparisons with LBNP 0 mmHg.

Fig 3 shows heart rate, MAP, stroke volume and cardiac output through the experiment. Stroke volume and cardiac output were reduced from baseline at all LBNP-levels. Heart rate was increased from LBNP 40 mmHg. MAP was not significantly different from baseline at any LBNP-level.

### ScO_2_, precerebral arteries and central hemodynamics

The precision of precerebral flow measurements was ±31 ml/min. As flow in the ICA was approximately four times flow in the VA, we also calculated the precision relative to each subject´s mean value, giving a precision of ±13%.

**Fig 4:**
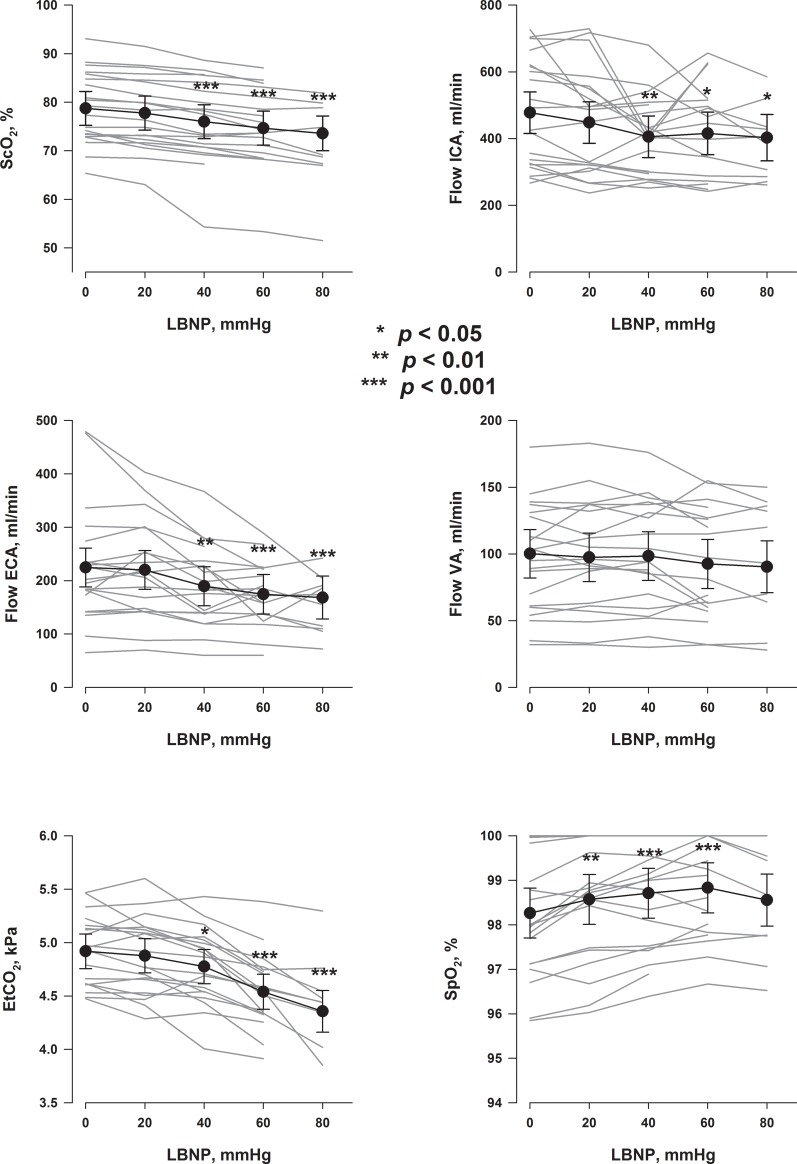
ScO_2_, precerebral blood flow, EtCO_2_ and SpO_2_. Grey lines are values for each subject. Black symbols are estimates with 95% CI for each LBNP-level. P values are for comparisons with LBNP 0 mmHg.

ScO_2_, EtCO_2_, SpO_2_ and blood flow in the ICA, ECA and VA are presented in Fig 4. ScO_2_ was reduced from LBNP 40 mmHg. Flow in both ICA and ECA were reduced from LBNP 40 mmHg whereas no change in VA flow was found for any LBNP-level. EtCO_2_ was reduced from LBNP 40 mmHg. A marginal increase in SpO_2_ was found from LBNP 20 mmHg to 60 mmHg.

**Fig 5:**
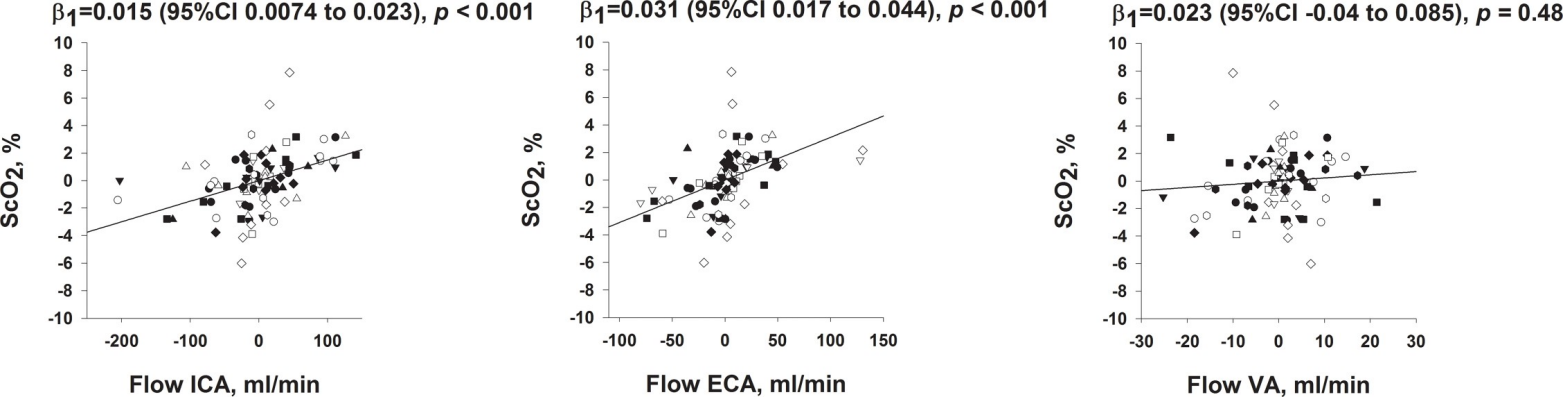
Scatterplots of ICA, ECA and VA flow vs. ScO_2_. Different subjects have different symbols. Each observation is the difference from that subject´s mean value, thus centering all values about 0. β_1_ is slope coefficient with confidence interval and p value, calculated with centered predictors.

Scatterplots of the within-subject changes in flow for the precerebral arteries vs. ScO_2,_ cardiac output and MAP are presented in Figs 5 and S1 Fig. Changes in flow in ICA and ECA were both associated with changes in ScO_2_ and cardiac output. There were no significant associations between changes in MAP and flow in any of the precerebral arteries. Cardiac output, ScO_2_ and EtCO_2_ were all associated with each other (S2 Fig).

### ScO_2_, precerebral arteries and skin blood flow

Scatterplots of the intra-individual changes in scalp skin blood flow and flow in the three precerebral arteries and ScO_2_ are presented in S3 Fig. Changes in flow in the VA were not associated with changes in scalp blood flow (lower or upper), and changes in ICA flow were not associated with changes in lower scalp blood flow. Otherwise, there were significant associations for the other comparisons. S4 Fig shows the significant associations between changes in acral (thumb) and scalp skin blood flow.

Respiratory rate was reduced from LBNP 40 mmHg (S5 Fig). There were no significant associations between changes in ScO_2_ or cardiac output and MAP (S6 Fig).

## Discussion

The main findings of the present study were that changes in ScO_2_ were associated with changes in blood flow in both ICA and ECA during LBNP. Changes in ScO_2_ were also associated with changes in cardiac output. Flow in both ICA and ECA were reduced with LBNP.

The association between ScO_2_ and cardiac output found in the present study (S2 Fig, left panel) could hold promise for the use of NIRS as a non-invasive tool to detect hypovolemia in unanesthetised patiens, e.g. trauma patients, although the estimated reduction in ScO_2_ was only 2.3% (95% CI 1.8 to 2.8) for a reduction in cardiac output of 1 l/min. The mechanism behind this association may however be of importance, and, to the best of our knowledge, this study adds new information regarding the mechanism of a ScO_2_-reduction in this situation.

After the seminal publication by Lassen [1] showing stable CBF between groups of patients with differing blood pressures above a certain level, CBF was assumed to be fairly constant, also within subjects, as long as blood pressure was kept within the autoregulatory blood pressure boundaries. The regulation of CBF is however complex, and CBF has been shown to decrease with decreasing cardiac output even if MAP is unaltered [2], compatible with the reduction in ICA flow with increasing LBNP found in the present study (Fig 4, upper right panel). This reduction in ICA-flow was associated with a reduction in ScO_2_ (Fig 5, left panel), suggesting that the reduced ScO_2_ could potentially be caused by reduced cerebral blood flow. However, we also found a reduction in ECA-flow with increasing LBNP (Fig 4, left middle panel) which was also associated with the change in ScO_2_ (Fig 5, middle panel). Cardiac output was associated with both the ICA and ECA-flow (S1 Fig). Extracranial contamination of ScO_2_ has been demonstrated [20], and it was therefore also possible that reduced ScO_2_ during hypovolemia was caused by reduced scalp perfusion, and not only reduced cerebral perfusion. However, both ICA and ECA-flow changes remained significant as explanatory variables of ScO_2_ changes in a multiple regression (Regressions 1 and 2 in S1 Appendix), suggesting that the observed ScO_2_ change could be caused by flow changes in both ICA and ECA.

We did not find a change in in the VA flow with hypovolemia. Although not a consistent finding [21, 22], these results agree with studies indicating regional differences between the areas supplied by the ICA and VA in response to hypovolemia with or without hypotension [13, 23–25], indicating that blood supply to the vital functions of the brainstem may be more strongly regulated. Blood flow in the VA was not associated with changes in ScO_2_ (Fig 5, right panel), consistent with the attachment of the ScO_2_-probes over the frontal region of the head reflecting circulation from the anterior and middle cerebral arteries [7]. Further, changes in VA-blood flow were not significantly associated with changes in cardiac output (S1 Fig, upper right panel).

CBF is affected by PaCO_2_, as hyperventilation leads to cerebral vasoconstriction [26]. In the present study, we measured EtCO_2_, but not PaCO_2_. EtCO_2_, cardiac output and ScO_2_ were all strongly associated (S2 Fig). This is consistent with EtCO_2_ reflecting reductions in cardiac output in preload changes [27], but the reduced ScO_2_ could also have been caused by reduced PaCO_2_ (reflected in reduced EtCO_2_), and not independently by cardiac output. However, when entering cardiac output and EtCO_2_ in a multiple regression (Regression 3 in S1 Appendix), both were significant predictors of ScO_2_, indicating independent effects on ScO_2_. Studies in animals and humans indicate that a decreased EtCO_2_ may not linearly reflect decreased PaCO_2_ during hypovolemia, as hypovolemia may induce pulmonary V/Q-mismatch, increased alveolar dead-space, and increased PaCO_2_—EtCO_2_-difference [28–31]. In the present study, respiratory rate was reduced with increasing hypovolemia (S5 Fig). We can only speculate on the reason for this reduction and if this is specific for the LBNP-model of hypovolemia, but increased inspiratory effort to increase venous return could have increased tidal volumes resulting in a compensatory reduction in respiratory rate. However, we did not measure tidal volume necessary to calculate minute ventilation. It is therefore difficult to state if or to what extent the measured reduction in EtCO_2_ reflected reduced PaCO_2_. In our model, we aimed to make the results valid for hypovolemic, spontaneously breathing patients and let any change in PaCO_2_ be similar to that occurring in real hypovolemia. Clamping CO_2_ in the present study could have eased the interpretation of the results, but would have rendered them clinically less meaningful as this study aimed to approximate the use of cerebral oximetry to diagnose hypovolemia in unanesthetised patients where CO_2_ is not clamped. It should also be noted, however, that hypovolemia and other stressors, e.g. pain, in a real trauma setting may induce hyperventilation to a larger extent than seen in our model. Future studies could potentially resolve these questions by measuring minute ventilation and PaCO_2,_ although the latter requires arterial catheterisation.

It has been speculated that the supraorbital skin area provides a window to the cerebral circulation, as the supraorbital artery is supplied from the anterior cerebral artery. We did however not find a significant association between LDF flow in supraorbital skin and blood flow in the ICA (S3 Fig, upper right panel). There was a significant association between changes in acral (thumb) and forehead skin blood flow (S4 Fig, left and middle panels), suggesting sympathetic vasoconstriction as a common mechanism. Although we did not find ScO_2_ to be reduced by pain elicited by the cold pressor test [32], one could therefore speculate that scalp skin blood flow is affected by sympathetic nervous activity, potentially confounding the relationship between volume status and ScO_2_. As previously shown, finger perfusion index is decreased with hypovolemia and was closely associated with acral LDF (S4 Fig, right panel) [32, 33].

We found a clinically marginal, but statistically significant increase in SpO_2_ on some LBNP-levels. We can only speculate if this represents a real change in arterial oxygen saturation or a measurement error in the pulse oximetry. For the purpose of this study, it was however important that there was no reduction in SpO_2_, as this could otherwise have indicated reduced arterial oxygen saturation as a possible mechanism for the reduction in ScO_2_.

### Methodological considerations

Doppler measurements of blood flow velocity depend on the angle of insonation, and small movements of the ultrasound probe may introduce large errors when the angle is high. We estimated precision of the ultrasound measurements before the LBNP-exposure, but small movements during increasing LBNP may increase the error.

Due to interference, it was not possible to measure the three precerebral arteries simultaneously. Measuring each artery over a long time could have made each measurement more precise, but In order for these measurements to be close in time and represent comparable hemodynamic conditions, the measurement of each artery was of a short duration.

We did not account for any change in vessel diameter at each LBNP-level in the calculations. A previous study showed that a head-up tilt reduced the ICA-diameter with 0.2 mm, but not the VA diameter [24]. In a LBNP-study on 10 subjects, the ICA diameter was only reduced by 0.3 mm at LBNP 50 mmHg, and the VA-diameter was not significantly reduced at any LBNP-level [25]. In the present study, post-hoc analyses revealed a statistically significant reduction in the ECA-diameter at LBNP 80 mmHg by 0.37 mm (95% CI 0.05 to 0.69, p = 0.016), but otherwise no significant changes in vessel diameter. As the Doppler-velocity measurements were obtained with the vessels imaged longitudinally, it is easy to foreshorten the diameter of individual measurements. As an example, given mean ICA-diameter of 6.0 mm, a measurement error of 0.5 mm to 6.5 mm would give 17% error in calculated flow. We therefore chose not to correct for the measured diameters, as the primary aim of our study was to compare associations of within-subject changes in precerebral flow and ScO_2_, and the increased accuracy of correcting for diameter would probably have been offset by a reduced precision. Analyses of the major results presented in Fig 5 were however also performed on values corrected for the measured diameters (S2 Appendix), but did not lead to results significantly different from the ones presented and the same conclusions would be drawn. Likewise, aortic diameter for calculation of stroke volume was assumed. Although absolute values may have been affected, this assumption would not affect our main analyses which are based on within-subject changes.

In the present study, MAP was not reduced before decompensation, and there was no association between changes in cardiac output or ScO_2_ and MAP (S6 Fig). Typically in the LBNP-model and actual hypovolemia in unanesthetised, healthy humans, MAP is only significantly reduced at circulatory decompensation, underscoring the strong regulation of MAP within subjects [6]. As we had a restricted range of MAP-values, strong statistical associations with other variables were therefore not to be expected [34]. In the present study, we relieved the LBNP at signs or symptoms of impending circulatory decompensation, as the main clinical challenge is to detect a compensated volume loss. As decompensation occurs, the clinical diagnosis of hypovolemia is often all too obvious.

Although the close association between changes in cardiac output and ScO_2_ (S2 Fig, left panel) may look encouraging, future studies could further explore if cerebral NIRS may be of clinical value in diagnosing compensated hypovolemia in an environment disturbed by e.g. stress, temperature changes, pain, blood transfusions and supplemental oxygen.

In conclusion; in the LBNP-model of hypovolemia in healthy conscious subjects, a reduction in ScO_2_ was associated with a reduction in blood flow in both the ICA and the ECA. Thus, reduced ScO_2_ during compensated hypovolemia seems to reflect both a reduction in cerebral blood flow and contamination from extracranial tissues.

## Supporting information

S1 Fig ICA, ECA and VA flow vs. cardiac output and mean arterial pressure. (PDF)

S2 Fig Cardiac output, ScO_2_ and EtCO_2_. (PDF)

S3 Fig Forehead skin blood vs. precerebral blood flow and ScO_2_. (PDF)

S4 Fig Forehead and acral skin blood flow. (PDF)

S5 Fig Respiratory rate. (PDF)

S6 Fig MAP vs. cardiac output and ScO_2_ vs. MAP. (PDF)

S1 Appendix Multiple regressions. (PDF)

S2 Appendix Changes in ScO2 vs. changes in blood flow in precerebral arteries corrected for measured diameter. (PDF)

## References

[1] Lassen NA. Cerebral blood flow and oxygen consumption in man. Physiol Rev. 1959;39(2):183–238. 10.1152/physrev.1959.39.2.183 13645234

[2] Meng L, Hou W, Chui J, Han R, Gelb AW. Cardiac Output and Cerebral Blood Flow: The Integrated Regulation of Brain Perfusion in Adult Humans. Anesthesiology. 2015;123(5):1198–208. 10.1097/ALN.0000000000000872 26402848

[3] Levine BD, Giller CA, Lane LD, Buckey JC, Blomqvist CG. Cerebral versus systemic hemodynamics during graded orthostatic stress in humans. Circulation. 1994;90(1):298–306. 8026012 10.1161/01.cir.90.1.298

[4] Brown CM, Dütsch M, Hecht MJ, Neundörfer B, Hilz MJ. Assessment of cerebrovascular and cardiovascular responses to lower body negative pressure as a test of cerebral autoregulation. Journal of the neurological sciences. 2003;208(1–2):71–8. 12639728 10.1016/s0022-510x(02)00438-0

[5] Ogoh S, Brothers RM, Barnes Q, Eubank WL, Hawkins MN, Purkayastha S, et al The effect of changes in cardiac output on middle cerebral artery mean blood velocity at rest and during exercise. J Physiol. 2005;569(Pt 2):697–704. 10.1113/jphysiol.2005.095836 16210355 PMC1464249

[6] Cannon JW. Hemorrhagic Shock. New England Journal of Medicine. 2018;378(4):370–9. 10.1056/NEJMra1705649 29365303

[7] Scheeren TW, Schober P, Schwarte LA. Monitoring tissue oxygenation by near infrared spectroscopy (NIRS): background and current applications. J Clin Monit Comput. 2012;26(4):279–87. 10.1007/s10877-012-9348-y 22467064 PMC3391360

[8] Sorensen H, Rasmussen P, Sato K, Persson S, Olesen ND, Nielsen HB, et al External carotid artery flow maintains near infrared spectroscopy-determined frontal lobe oxygenation during ephedrine administration. Br J Anaesth. 2014;113(3):452–8. 10.1093/bja/aet481 24508985

[9] Davie SN, Grocott HP. Impact of extracranial contamination on regional cerebral oxygen saturation: a comparison of three cerebral oximetry technologies. Anesthesiology. 2012;116(4):834–40. 10.1097/ALN.0b013e31824c00d7 22343469

[10] Sorensen H, Secher NH, Siebenmann C, Nielsen HB, Kohl-Bareis M, Lundby C, et al Cutaneous vasoconstriction affects near-infrared spectroscopy determined cerebral oxygen saturation during administration of norepinephrine. Anesthesiology. 2012;117(2):263–70. 10.1097/ALN.0b013e3182605afe 22739762

[11] Cooke WH, Ryan KL, Convertino VA. Lower body negative pressure as a model to study progression to acute hemorrhagic shock in humans. J Appl Physiol (1985). 2004;96(4):1249–61. 15016789 10.1152/japplphysiol.01155.2003

[12] Johnson BD, van Helmond N, Curry TB, van Buskirk CM, Convertino VA, Joyner MJ. Reductions in central venous pressure by lower body negative pressure or blood loss elicit similar hemodynamic responses. J Appl Physiol (1985). 2014;117(2):131–41. 24876357 10.1152/japplphysiol.00070.2014 PMC4459917

[13] Kay VL, Rickards CA. The role of cerebral oxygenation and regional cerebral blood flow on tolerance to central hypovolemia. Am J Physiol Regul Integr Comp Physiol. 2016;310(4):R375–83. 10.1152/ajpregu.00367.2015 26676249

[14] Schoning M, Scheel P. Color duplex measurement of cerebral blood flow volume: intra- and interobserver reproducibility and habituation to serial measurements in normal subjects. J Cereb Blood Flow Metab. 1996;16(3):523–31. 10.1097/00004647-199605000-00020 8621758

[15] Eriksen M, Walløe L. Improved method for cardiac output determination in man using ultrasound Doppler technique. Medical & Biological Engineering & Computing. 1990;28(6):555–60. 2287179 10.1007/BF02442607

[16] Pinheiro J, Bates D, DebRoy S, Sarkar D, Team RC. nlme: Linear and nonlinear mixed effects models (R package version 3.1–128, 2016). R software. 2017.

[17] Hothorn T, Bretz F, Westfall P. Simultaneous inference in general parametric models. Biom J. 2008;50(3):346–63. 10.1002/bimj.200810425 18481363

[18] van de Pol M, Wright J. A simple method for distinguishing within- versus between-subject effects using mixed models. Animal Behaviour. 2009;77(3):753–8.

[19] Bland JM, Altman DG. Measuring agreement in method comparison studies. Stat Methods Med Res. 1999;8(2):135–60. 10.1177/096228029900800204 10501650

[20] Sorensen H. Near infrared spectroscopy evaluated cerebral oxygenation during anesthesia. Dan Med J. 2016;63(12). 27910802

[21] Tymko MM, Rickards CA, Skow RJ, Ingram-Cotton NC, Howatt MK, Day TA. The effects of superimposed tilt and lower body negative pressure on anterior and posterior cerebral circulations. Physiol Rep. 2016;4(17). 10.14814/phy2.12957 PMC5027361 27634108

[22] Deegan BM, Cooke JP, Lyons D, ÓLaighin G, Serrador JM. Cerebral autoregulation in the vertebral and middle cerebral arteries during combine head upright tilt and lower body negative pressure in healthy humans. Engineering in Medicine and Biology Society (EMBC), 2010 Annual International Conference of the IEEE. 2010:2505–8. 10.1109/IEMBS.2010.5626647 21096171

[23] Lewis NC, Smith KJ, Bain AR, Wildfong KW, Numan T, Ainslie PN. Impact of transient hypotension on regional cerebral blood flow in humans. Clin Sci (Lond). 2015;129(2):169–78. 25697830 10.1042/CS20140751

[24] Sato K, Fisher JP, Seifert T, Overgaard M, Secher NH, Ogoh S. Blood flow in internal carotid and vertebral arteries during orthostatic stress. Exp Physiol. 2012;97(12):1272–80. 10.1113/expphysiol.2012.064774 22689443

[25] Ogoh S, Sato K, Okazaki K, Miyamoto T, Hirasawa A, Sadamoto T, et al Blood flow in internal carotid and vertebral arteries during graded lower body negative pressure in humans. Exp Physiol. 2015;100(3):259–66. 10.1113/expphysiol.2014.083964 25641216

[26] Meng L, Gelb AW. Regulation of cerebral autoregulation by carbon dioxide. Anesthesiology. 2015;122(1):196–205. 10.1097/ALN.0000000000000506 25401418

[27] Lakhal K, Nay MA, Kamel T, Lortat-Jacob B, Ehrmann S, Rozec B, et al Change in end-tidal carbon dioxide outperforms other surrogates for change in cardiac output during fluid challenge. Br J Anaesth. 2017;118(3):355–62. 10.1093/bja/aew478 28186263

[28] Isserles SA, Breen PH. Can changes in end-tidal PCO2 measure changes in cardiac output? Anesth Analg. 1991;73(6):808–14. 1952183 10.1213/00000539-199112000-00023

[29] Garnett AR, Glauser FL, Ornato JP. Hypercarbic arterial acidemia following resuscitation from severe hemorrhagic shock. Resuscitation. 1989;17(1):55–61. 2538901 10.1016/0300-9572(89)90079-8

[30] Dubin A, Murias G, Estenssoro E, Canales H, Sottile P, Badie J, et al End-tidal CO2 pressure determinants during hemorrhagic shock. Intensive Care Med. 2000;26(11):1619–23. 11193267 10.1007/s001340000669

[31] Dowell AR, Schmid PG, Nutter DO, Sullivan KN. Ventilation, lung volumes, and gas exchange during lower body negative pressure. J Appl Physiol. 1969;26(3):352–9. 10.1152/jappl.1969.26.3.352 5773177

[32] Hoiseth LO, Hisdal J, Hoff IE, Hagen OA, Landsverk SA, Kirkeboen KA. Tissue oxygen saturation and finger perfusion index in central hypovolemia: influence of pain. Crit Care Med. 2015;43(4):747–56. 10.1097/CCM.0000000000000766 25513787

[33] van Genderen ME, Bartels SA, Lima A, Bezemer R, Ince C, Bakker J, et al Peripheral perfusion index as an early predictor for central hypovolemia in awake healthy volunteers. Anesth Analg. 2013;116(2):351–6. 10.1213/ANE.0b013e318274e151 23302972

[34] Bland JM, Altman DG. Correlation in restricted ranges of data. BMJ. 2011;342:d556 10.1136/bmj.d556 21398359

