# Correction: Associations between changes in precerebral blood flow and cerebral oximetry in the lower body negative pressure model of hypovolemia in healthy volunteers

**DOI:** 10.1371/journal.pone.0220403

**Published:** 2019-07-23

**Authors:** Jonny Hisdal, Svein Aslak Landsverk, Ingrid Elise Hoff, Ove Andreas Hagen, Knut Arvid Kirkebøen, Lars Øivind Høiseth

Fig 2, “Measurements,” appears as a duplicate of Fig 1. Please view the correct Fig 2 here.

**Fig 2:**
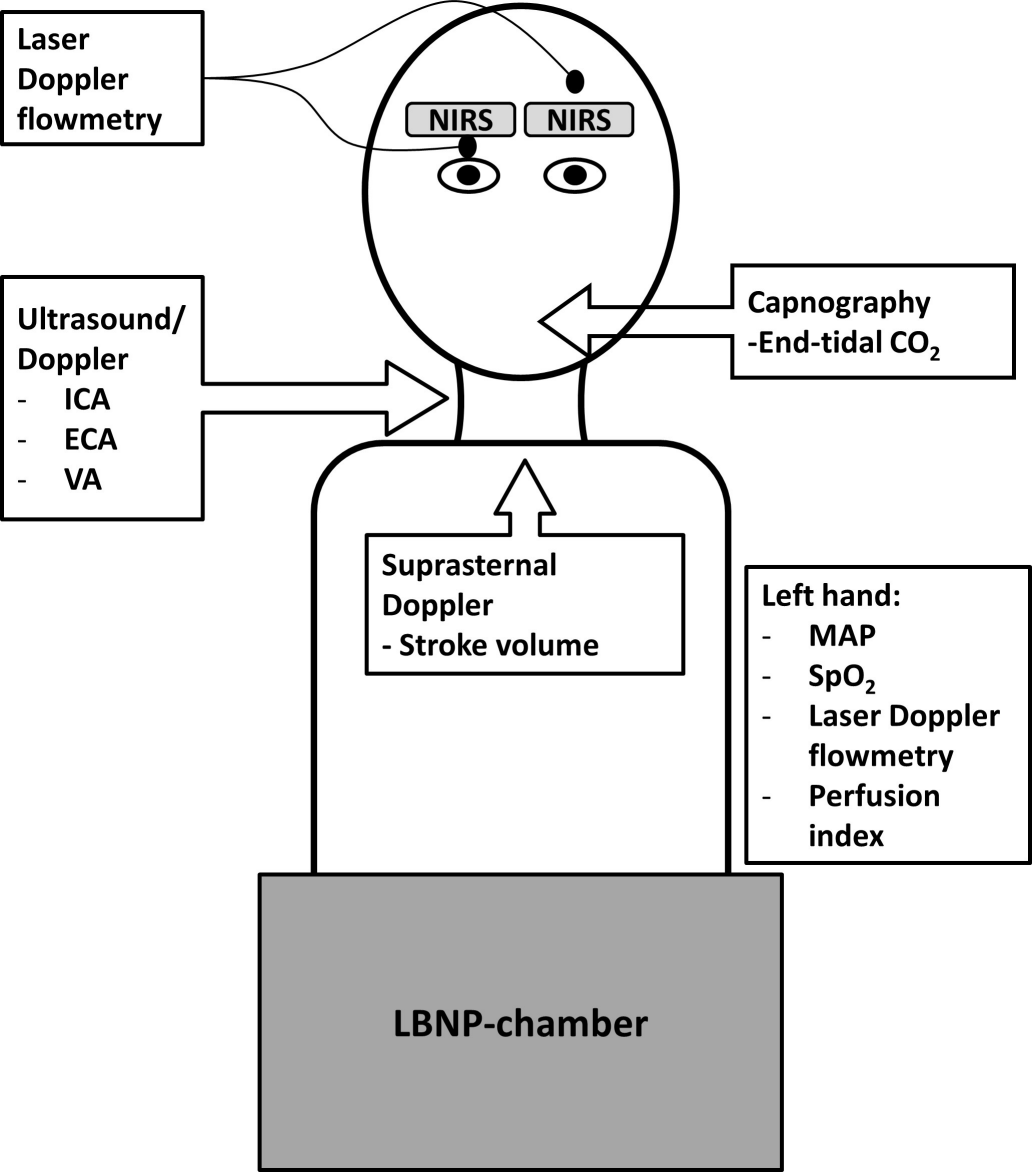
Measurements. Schematic overview of the performed measurements.
