# Supplementary material for: Associations between changes in precerebral blood flow and cerebral oximetry in the lower body negative pressure model of hypovolemia in healthy volunteers
Source: PLoS One. 2019 Jun 28;14(6):e0219154. doi: 10.1371/journal.pone.0219154 (PMC6599124; doi:10.1371/journal.pone.0219154)
Supplement: S2 Fig — (PDF) [file pone.0219154.s002.pdf]

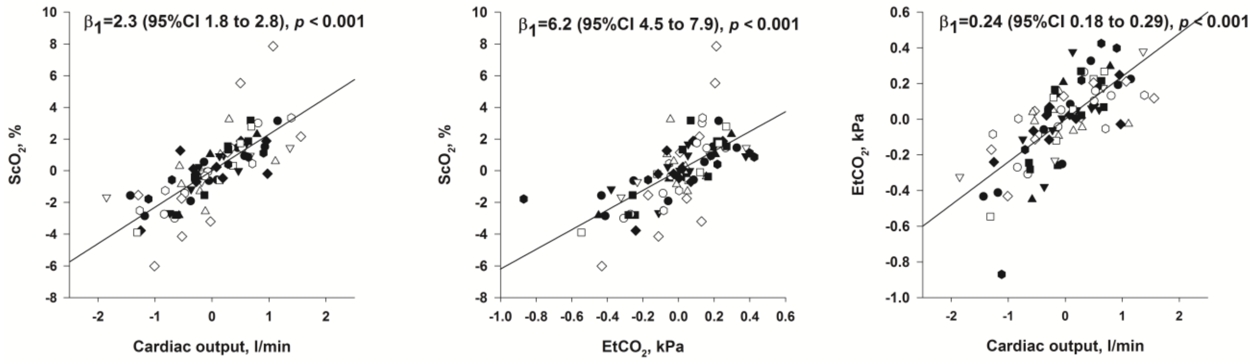

S2 Fig. Cardiac output, ScO_2 and $EtCO_2$. Different subjects have different symbols. Each observation is the difference from that subject´s mean value, thus centering all values about 0. β_1 is slope coefficient with confidence interval and p value, calculated with centered predictors.
