# Supplementary material for: Associations between changes in precerebral blood flow and cerebral oximetry in the lower body negative pressure model of hypovolemia in healthy volunteers
Source: PLoS One. 2019 Jun 28;14(6):e0219154. doi: 10.1371/journal.pone.0219154 (PMC6599124; doi:10.1371/journal.pone.0219154)
Supplement: S3 Fig — (PDF) [file pone.0219154.s003.pdf]

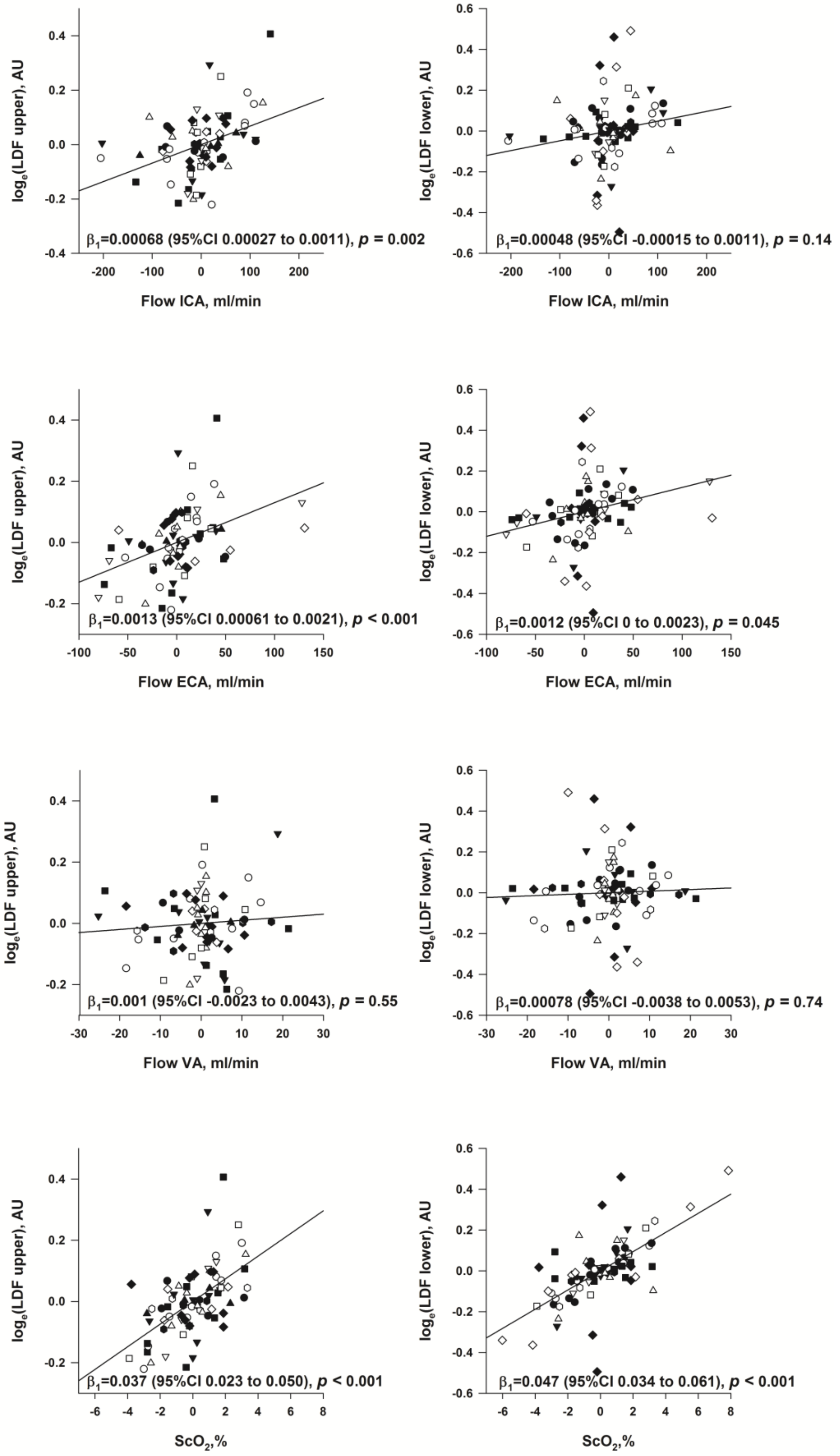

log_e(LDF upper), AU
0.4
0.2
0.0
-0.2
-0.4
β1=0.00068 (95%CI 0.00027 to 0.0011), p = 0.002
-200
-100
0
100
200
Flow ICA, ml/min
log_e(LDF lower), AU
0.6
0.4
0.2
0.0
-0.2
-0.4
-0.6
β1=0.00048 (95%CI -0.00015 to 0.0011), p = 0.14
Flow ICA, ml/min
log_e(LDF upper), AU
β1=0.0013 (95%CI 0.00061 to 0.0021), p < 0.001
-100
-50
0
50
100
150
Flow ECA, ml/min
log_e(LDF lower), AU
β1=0.0012 (95%CI 0 to 0.0023), p = 0.045
Flow ECA, ml/min
log_e(LDF upper), AU
β1=0.001 (95%CI -0.0023 to 0.0043), p = 0.55
-30
-20
-10
0
10
20
30
Flow VA, ml/min
log_e(LDF lower), AU
β1=0.00078 (95%CI -0.0038 to 0.0053), p = 0.74
Flow VA, ml/min
log_e(LDF upper), AU
β1=0.037 (95%CI 0.023 to 0.050), p < 0.001
-6
-4
-2
0
2
4
6
8
ScO2,%
log_e(LDF lower), AU
β1=0.047 (95%CI 0.034 to 0.061), p < 0.001
ScO2,%

S3 Fig. Forehead skin blood vs. precerebral blood flow and ScO_2. Scatterplots of changes in skin blood flow measured by laser Doppler flowmetry (LDF, $\log_e$ transformed) high in the forehead (LDF upper) and supraorbital (LDF lower) vs. precerebral blood flow and ScO_2. Each observation is the difference from that subject´s mean value, thus centering all values about 0. β_1 is slope coefficient with confidence interval and p value, calculated with centered predictors.
