# Supplementary material for: Associations between changes in precerebral blood flow and cerebral oximetry in the lower body negative pressure model of hypovolemia in healthy volunteers
Source: PLoS One. 2019 Jun 28;14(6):e0219154. doi: 10.1371/journal.pone.0219154 (PMC6599124; doi:10.1371/journal.pone.0219154)
Supplement: S4 Fig — (PDF) [file pone.0219154.s004.pdf]

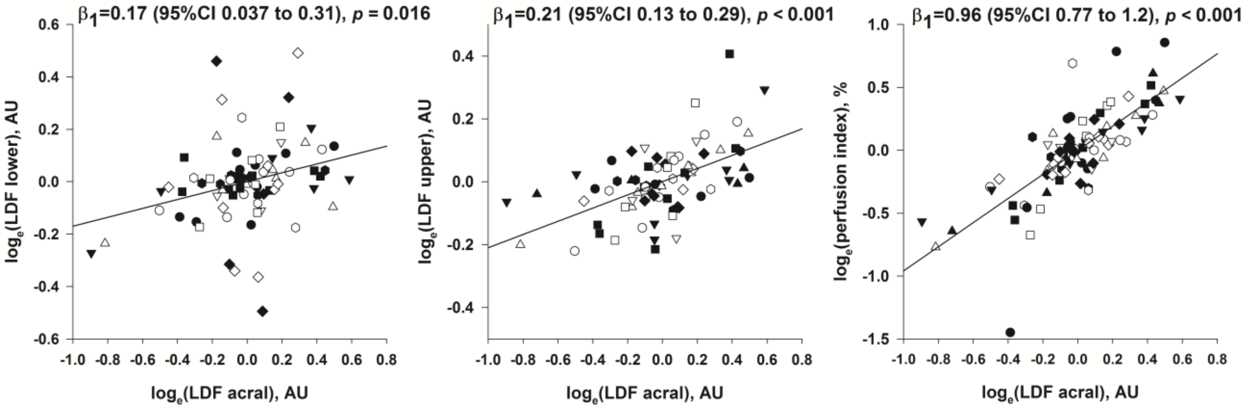

S4 Fig. Forehead and acral skin blood flow. Changes in lower and upper forehead skin blood flow vs. acral (thumb) skin blood flow measured by laser Doppler flowmetry (LDF, left and middle panels) and finger perfusion index vs. acral (thumb) skin blood flow (right panel). All values are $\log_e$-transformed. Each observation is the difference from that subject´s mean value, thus centering all values about 0. β_1 is slope coefficient with confidence interval and *p* value, calculated with centered predictors.
