# Supplementary material for: Associations between changes in precerebral blood flow and cerebral oximetry in the lower body negative pressure model of hypovolemia in healthy volunteers
Source: PLoS One. 2019 Jun 28;14(6):e0219154. doi: 10.1371/journal.pone.0219154 (PMC6599124; doi:10.1371/journal.pone.0219154)
Supplement: S5 Fig — (PDF) [file pone.0219154.s005.pdf]

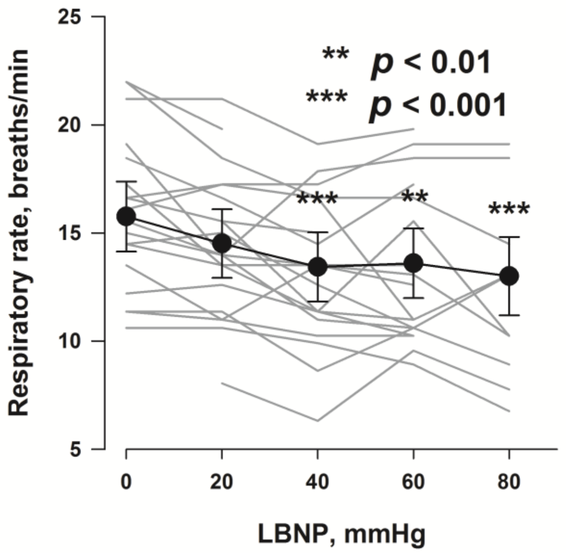

S5 Fig. Respiratory rate. Grey lines are values for each subject. Black symbols are estimates with 95% CI for each LBNP-level. P values are for comparisons with LBNP 0 mmHg. LBNP, lower body negative pressure.
