# Supplementary material for: Associations between changes in precerebral blood flow and cerebral oximetry in the lower body negative pressure model of hypovolemia in healthy volunteers
Source: PLoS One. 2019 Jun 28;14(6):e0219154. doi: 10.1371/journal.pone.0219154 (PMC6599124; doi:10.1371/journal.pone.0219154)
Supplement: S1 Appendix — (PDF) [file pone.0219154.s007.pdf]

Regressions

All regressions are performed in a mixed model, random intercept, with subjects as random effect. Only the fixed effects are presented. All predictors are calculated as differences of each observation (LBNP-level) from that subject´s mean value.

Regression 1. ScO_2 regressed on ICA, ECA and VA.

Dependent variable:

ScO_2 (%)

Predictors:

Centered changes in flow ICA (ml/min) +
Centered changes in flow ECA (ml/min) +
Centered changes in flow VA (ml/min)

	Value	Std.Error	DF	t-value	*p* value
Intercept	77	1.8	63	43	<0.001
Flow ICA	0.0094	0.0042	63	2.2	0.031
Flow ECA	0.023	0.0078	63	2.9	0.0049
Flow VA	0.006	0.03	63	0.2	0.84

Regression 2. ScO_2 regressed on ICA and ECA.

Dependent variable:

ScO_2 (%)

Predictors:

Centered changes in flow ICA (ml/min) +
Centered changes in flow ECA (ml/min)

	Value	Std.Error	DF	t-value	*p* value
Intercept	77	1.8	64	43	<0.001
Flow ICA	0.0095	0.0042	64	2.3	0.027
Flow ECA	0.023	0.0077	64	2.9	0.0046

Regression 3. ScO_2 regressed on CO and $EtCO_2$.

Dependent variable:

ScO_2 (%)

Predictors:

Centered changes in CO (l/min) +

Centered changes in $EtCO_2$ (kPa)

	Value	Std.Error	DF	t-value	*p* value
Intercept	77	1.8	64	43	<0.001
Cardiac output	1.8	0.35	64	5.2	<0.001
$EtCO_2$	2.2	1.1	64	2.0	0.048
