# Supplementary material for: Associations between changes in precerebral blood flow and cerebral oximetry in the lower body negative pressure model of hypovolemia in healthy volunteers
Source: PLoS One. 2019 Jun 28;14(6):e0219154. doi: 10.1371/journal.pone.0219154 (PMC6599124; doi:10.1371/journal.pone.0219154)
Supplement: S2 Appendix — (PDF) [file pone.0219154.s008.pdf]

Diameter, precerebral arteries

LBNP-level, mmHg	0	20	40	60	80
**Internal carotid artery, mm**	5.5 (5.1 to 6.0)	5.4 (5.0 to 5.8), p = 0.52	5.4 (5.0 to 5.9), p = 0.70	5.3 (4.9 to 5.8), p = 0.29	5.4 (4.9 to 5.8), p = 0.44
**External carotid artery, mm**	4.2 (3.9 to 4.6)	4.2 (3.8 to 4.5), p = 0.88	4.0 (3.7 to 4.4), p = 0.19	4.0 (3.6 to 4.3), p = 0.050	3.9 (3.5 to 4.3), p = 0.016
**Vertebral artery, mm**	3.3 (3.0 to 3.6)	3.4 (3.1 to 3.6), p = 0.35	3.3 (3.0 to 3.6), p = 0.93	3.3 (3.0 to 3.6), p = 0.93	3.3 (3.0 to 3.6), p = 0.98

Estimates (95% CI) of diameters of the precerebral arteries from linear mixed regression models with subjects as random effects. P values are for comparisons to LBNP 0 mmHg.

Regression slopes, flow corrected for the measured diameter at each LBNP-level

The results below correspond to the uncorrected values presented in Fig 4.

Slope of ScO_2 regressed on corrected flow, internal carotid artery:

0.013 (95%CI 0.0069 to 0.020), p < 0.001

Slope of ScO_2 regressed on corrected flow, external carotid artery:

0.024 (95%CI 0.013 to 0.036), p < 0.001

Slope of ScO_2 regressed on corrected flow, vertebral artery:

0.029 (95%CI -0.0098 to 0.068), P=0.15
